# Quality as an organizational strategy: building a system of improvement

**DOI:** 10.3389/frhs.2026.1726688

**Published:** 2026-05-29

**Authors:** David M. Williams, Clifford L. Norman, Lloyd P. Provost

**Affiliations:** 1 DMW Austin, Austin, TX, United States; 2 Associates in Process Improvement, Austin, TX, United States

**Keywords:** healthcare, improvement science, leadership, organizational learning, quality improvement, quality strategy, systems thinking

## Abstract

Improvement science offers proven theories and methods that can be applied to specific projects, but they have rarely been applied to the leadership systems of healthcare organizations. This paper presents Quality as an Organizational Strategy (QOS), a method for building a system of improvement that embeds improvement science into leadership and strategy. QOS centers on five activities: defining and communicating purpose, viewing the organization as a system, building a system for obtaining information, planning to improve, and managing improvement efforts. Examples from healthcare settings illustrate how leaders use these activities to align vision, strategy, and culture while fostering continuous learning. QOS is designed to enable organizations to pursue achieving strategic aims and sustaining performance over time. By broadening project-based improvement to improving the organizations as a system, this paper expands the application of improvement science with a practical, generalizable approach for transforming complex health systems.

## Introduction

Healthcare organizations have created complex systems that must achieve and sustain results in patient outcomes, control costs, and deliver a patient-centered experience (1) while engaging a diverse workforce (2). Improvement science offers approaches to achieve these aims.

Improvement has evolved in healthcare over the last few decades (3, 4). Existing approaches typically apply improvement methods (e.g., flow diagrams, PDSA cycles, and time series data) to projects or functions at the point of care, such as reducing falls or sepsis. A national survey in the United States of health systems found that 69.3% reported adoption of improvement methods such as the Model for Improvement, Lean, or Six Sigma but just 12% reported system-wide impacts (5).

Examples exist of moving to considering systems as the focus of improvement strategy. Microsystems approaches expand from the specific care process to a surrounding subsystem where people work together to deliver care to a defined population of patients (6). Another example reveals that health systems have used systems thinking to work on achieving hospital-wide flow (7).

As noted earlier, only 12% of health systems reported improvement being applied across the organization in a mature manner. Chartering and executing improvement efforts across the organization does not mean that these theories and methods are applied in a systematic and integrated way to organizational-level functions. Deming noted success in adoption of improvement methods to specific processes and functions but estimated that this impacted only a small proportion of the opportunity in organizations (8). He predicted that significant progress could be made if leaders applied these theories and methods to core business systems and adopted a focus on quality as their strategy.

The methods of QOS described in this paper aim to meet the challenge of an integrated approach for leaders to build a system of improvement that continually enhances organizational performance over time. QOS creates a framework of leadership activities, and it describes how to integrate these activities. For example, how does establishing a purpose shape the understanding of the organization as a system? How does the planning process inform improvement and operations? This integration and interdependence are a novel characteristic not found in other organization-wide approaches.

Improvement science was first described as emerging across the 20th century and including the foundations recognized by Deming in his System of Profound Knowledge (9). The term is noted to be used under many operational definitions in the literature (10). Improvement science as described in this paper includes the interaction of systems thinking, understanding variation, psychology of change, and the theory of knowledge that are applied to improve the performance of processes, products, services, organizations, and communities. Their proper application requires integration of a set of improvement methods and tools with a knowledge of subject matter to develop, test, implement, and spread changes (11). This definition includes a foundational body of knowledge, a journey of improvement, and a focus on outcomes.

Several approaches exist to address quality across an organization, including the Toyota Production System (12), Lean (13), the Juran Trilogy (14) and adaptations (15), and the learning health system (16). There are also many award programs that address the whole organization, including the Malcolm Baldrige National Quality Award, the Deming Prize, the Shingo Prize, and external assessment organizations and accreditation bodies.

**Figure 1:**
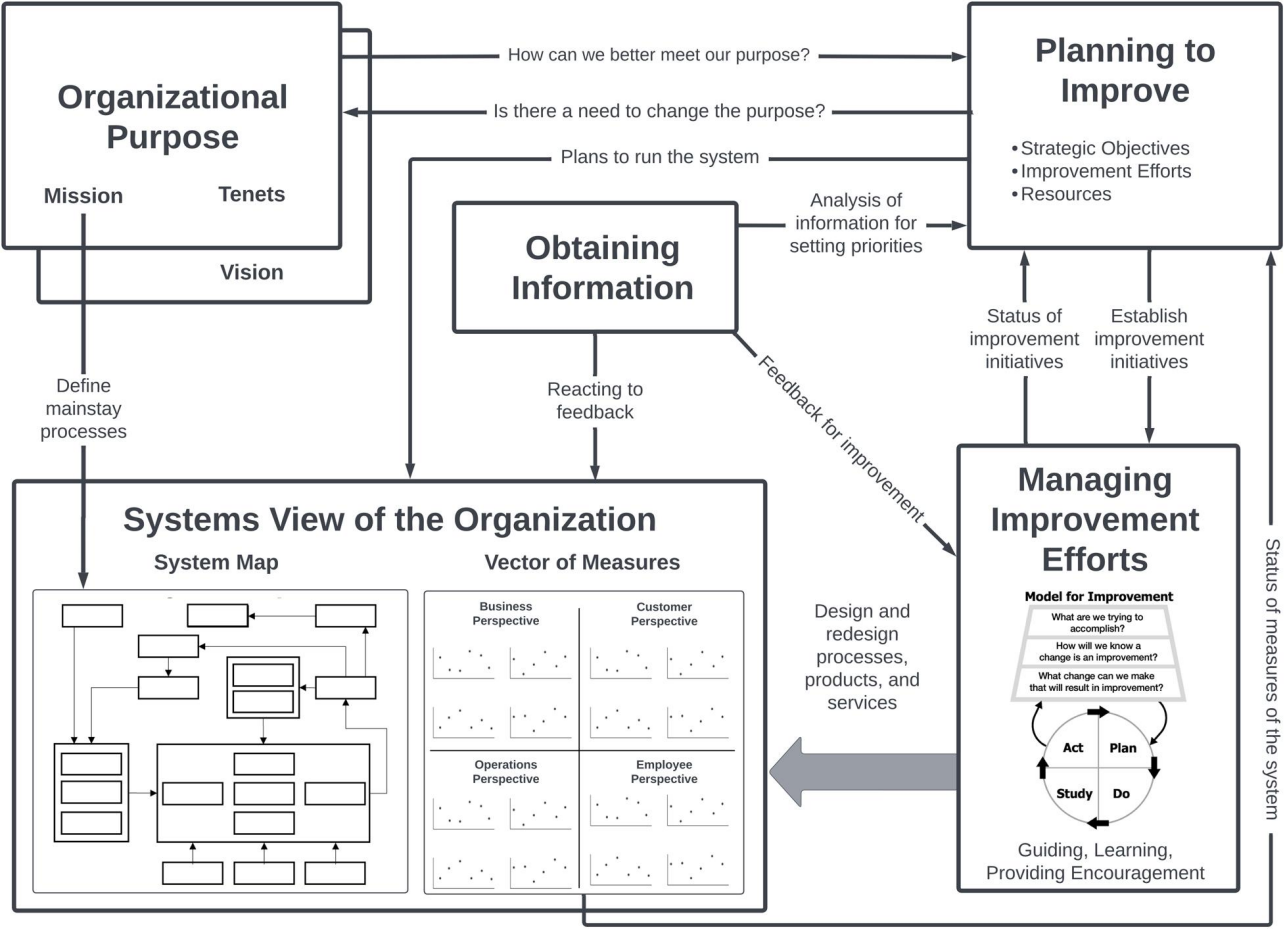
Quality as an organizational strategy: activities for leaders to build a system of improvement. This is a conceptual diagram showing the five interrelated QOS activities and their relationships.

QOS is unique in that it incorporates methods from each of the four foundational bodies of knowledge of improvement science and the framework facilitates the integration and interdependencies of the activities as a system. This paper describes QOS as a system of five interrelated activities for leaders (Figure 1). QOS forms a practical method for building a system of improvement that integrates leadership, rigorous improvement, and people engagement (11). QOS can be adopted in any organization, but this paper focuses on applications exclusive to health and healthcare, such as healthcare delivery organizations, public health, and other complex health systems. QOS supports leaders to adopt quality as a strategy (17) and continually improve quality across the whole system (15).

The QOS framework can be implemented at different degrees of rigor and sophistication based on the leadership, the organization's current state, local context, and preferences of the leaders and staff. For example, how QOS is implemented in community primary care clinic will vary from how a large, multihospital academic health system approaches pursuing quality as their strategy.

## Methods

The objective of QOS is to build a system of organization-wide improvement based on the theories and methods of improvement science. The methods are designed to enable leaders to foster a learning organization (18) that develops, gathers, and shares knowledge and adapts its behaviors and practices. The concept of viewing an organization as a production system was first outlined by Deming in 1950 (19). Associates in Process Improvement began to develop a framework based on Deming's concept and theories in the 1980s (11). The strategy included three basic elements:

1. A foundation of continuous matching of products and services to the need through design and redesign of processes, products, and services.
2. An organization that performs as a system to achieve this, focusing on the need.
3. A set of methods to ensure that changes result in real improvement of the organization.

The framework of activities evolved through application with leaders and organizations. Over nearly 40 years, the methods have been tested and adapted in diverse organizations inside and outside of healthcare. The documentation of the QOS methods first appeared in a workbook in 1996 and later as a chapter in the first edition of *The Improvement Guide* (9).

Table 1 outlines the five methods of QOS and describes actions for leaders to make quality an organizational strategy (11).

**Figure 2:**
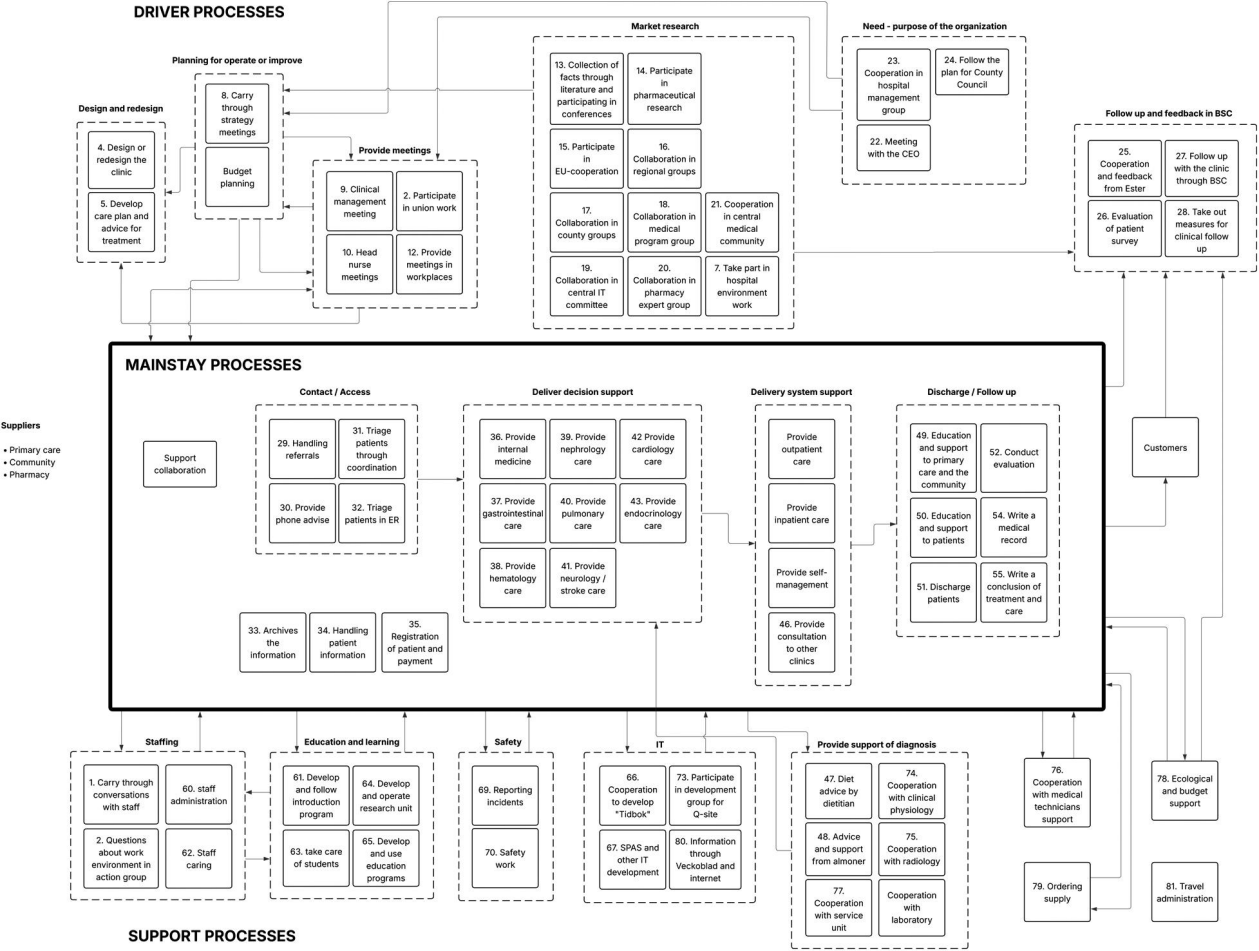
System map of a department of internal medicine depicting mainstay (middle bold box), driver (top), and support (bottom) processes and linkages reflecting interdependencies (Jönköping County Council, Sweden). Each individual numbered box is a process. Clusters of related processes are framed with a dashed box. The arrows depict the flow and interrelationship of processes. Reprinted with permission from Region Jönköping County, Göran Henriks. Permission granted 15 October 2025.

**Figure 3:**
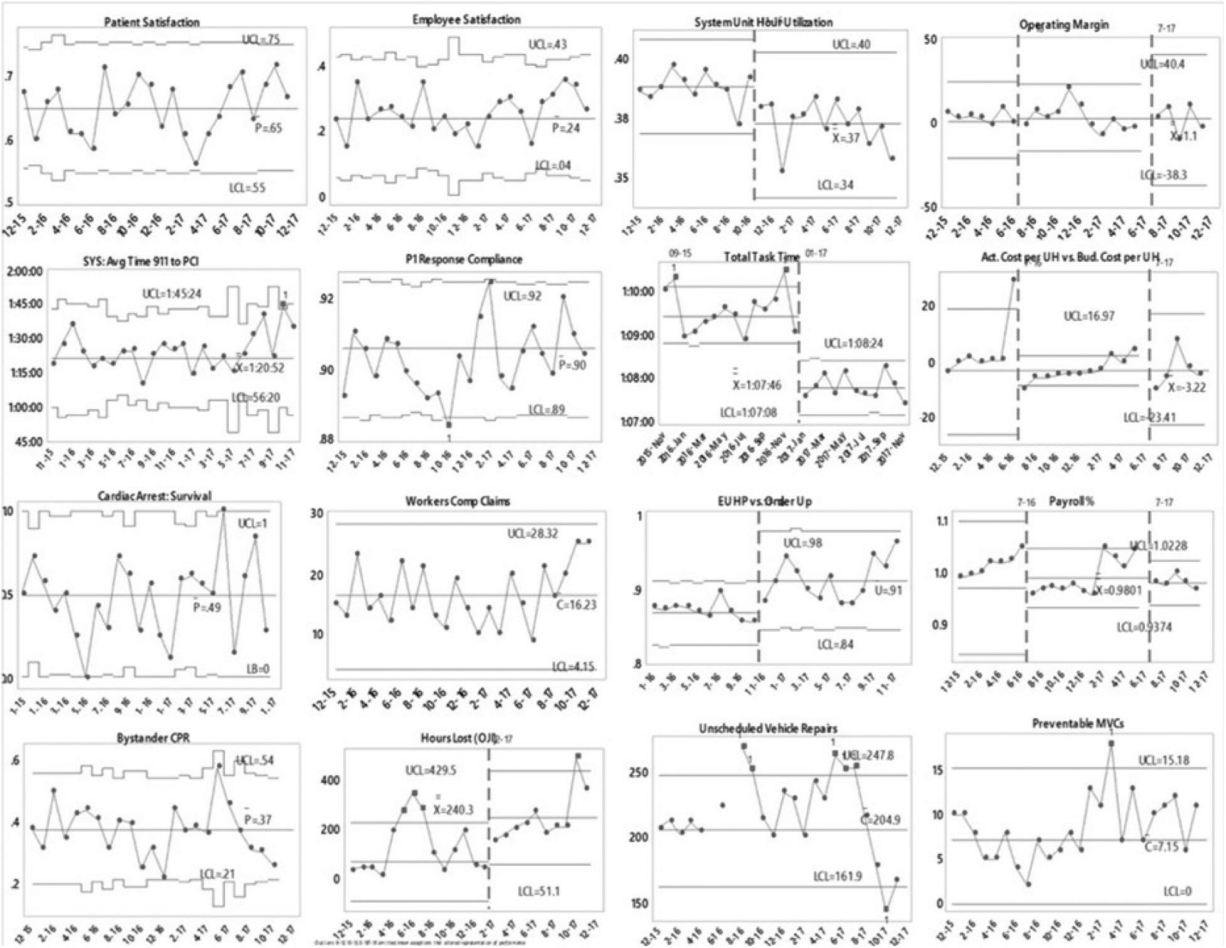
Vector of measures displayed in Shewhart charts from an ambulance service. Each chart is an outcome or key process measure depicting different perspectives of the system (Mecklenburg EMS Agency, Charlotte, USA).

**Figure 4:**
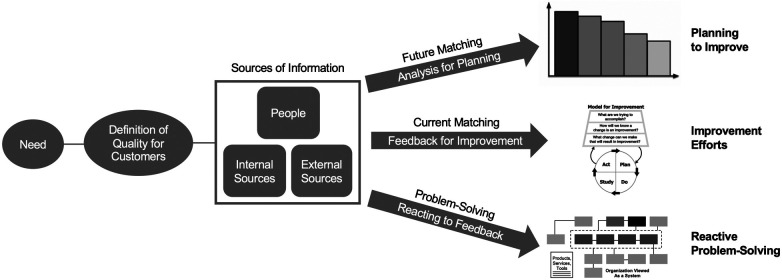
System for gathering information. This is a conceptual diagram illustrating how information flows from diverse sources to support problem-solving, improvement, and planning.

**Figure 5:**
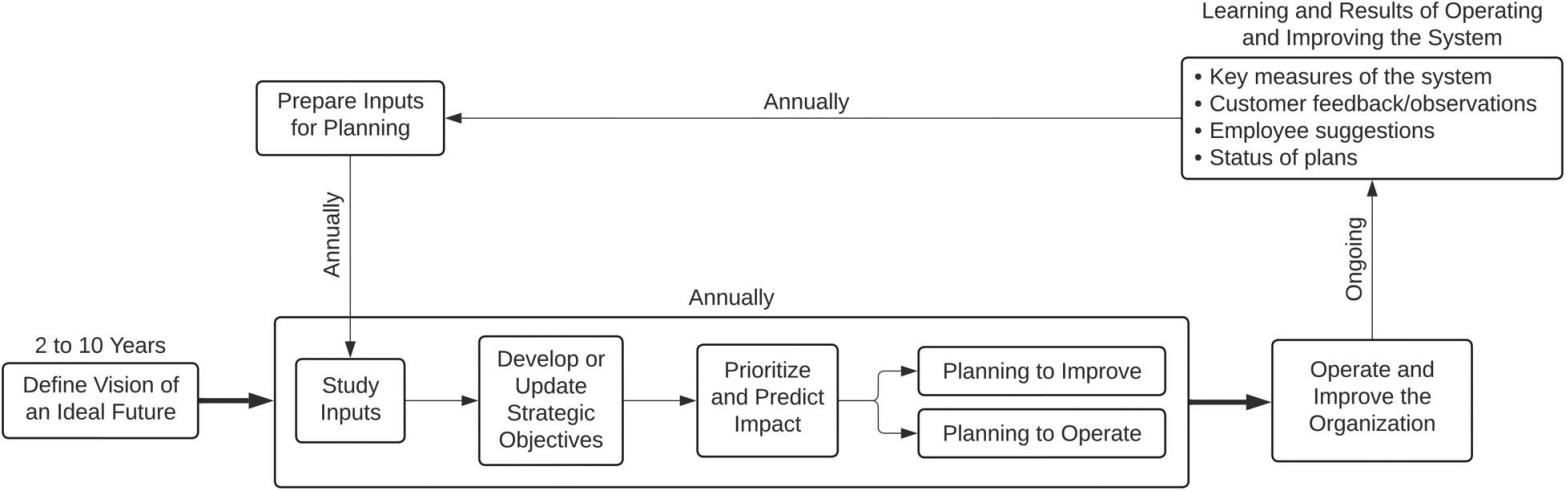
Planning as a system. This is a conceptual diagram showing the planning process and its inputs, cycles, and time frames.

**Figure 6:**
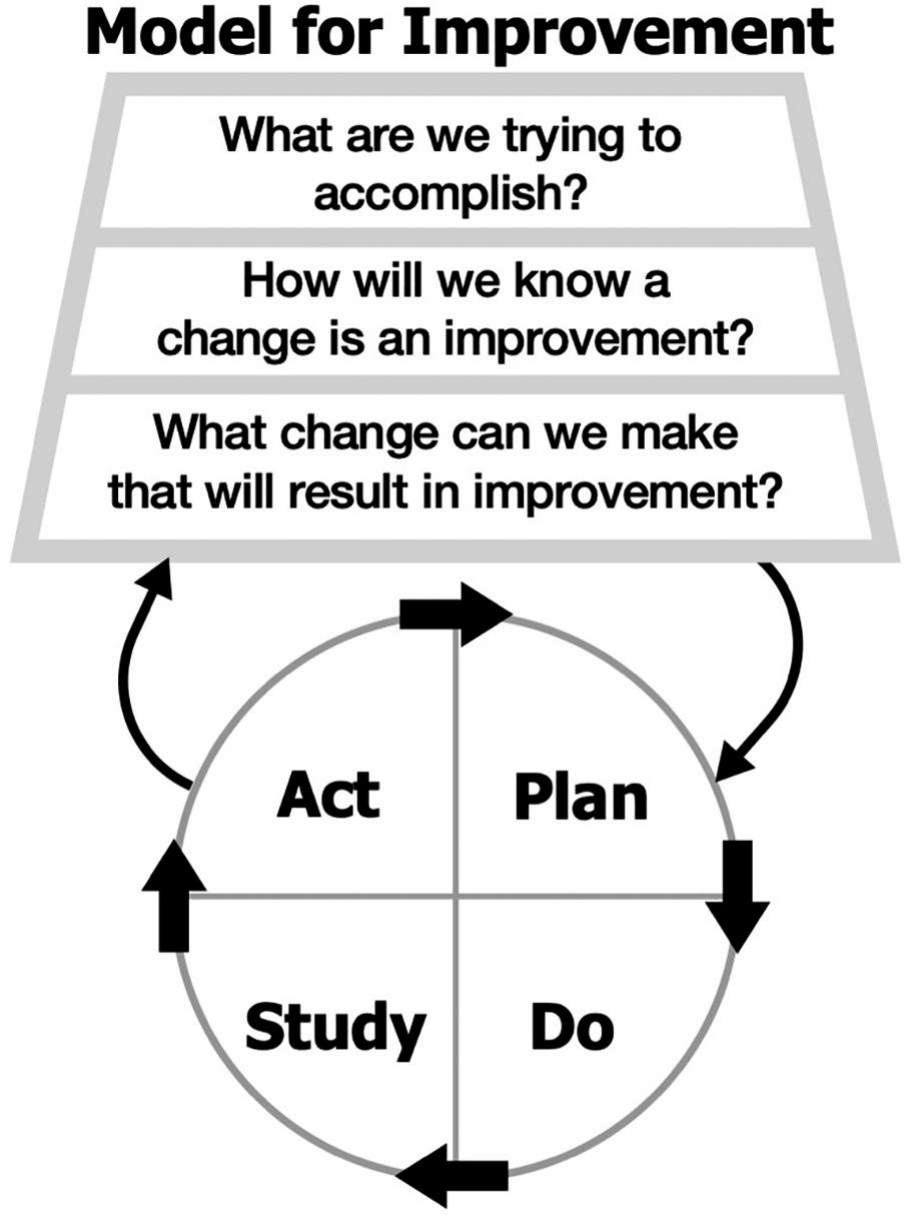
Model for improvement. This is a conceptual diagram showing the three questions and Plan–Do–Study–Act cycle used in QOS improvement efforts.

1. **Establishing and communicating purpose**—Organizations are established to provide products and services that match a need in society (20). What are clinicians and staff in healthcare trying to do? A family medicine practice is providing service to meet a need for healthcare through prevention and treatment. The practice may offer telehealth as a service for seeing your physician assistant. The service is driven because leaders at the practice discovered that patients experienced delays in seeking care because of difficulties of making time to visit in person.
  Understanding the need is useful for clarifying the purpose of the organization including its mission, vision, and tenets (11). The mission describes the contribution of the organization and the need it intends to fulfill in society. The vision frames how the organization will change in the future to better match the need. Tenets are how leaders and staff conduct themselves and play a role in culture. An organization's purpose is used by leaders to communicate with all stakeholders and support decisions, planning, and innovation. Table 2 is an example of a purpose statement from Cincinnati Children's Medical Center (21).
2. **Viewing the organization as a system**—Creating a systems view of an organization includes two methods: a system map and a vector of measures. These methods are designed to enable leaders to see their organization as a system by bringing together two foundational lenses of improvement science: appreciation of a system and understanding variation.
  *System map*. Developing a system map that visually displays work as a constellation of interdependent processes. All work can be described as a process. Flow diagrams are used to describe the steps of a process (22, 23). In organizations, processes do not act in isolation (24). A process may be dependent on inputs from another process and the output, or results, of a process may be important to another team and their process. A system map is a method to visualize key processes of an organization and their interdependence (11). Like maps used in geography, system maps can depict different levels of detail: overall organizational, a location or service line, or a department or unit.
  Figure 2 is a system map of the internal medicine department from the healthcare system in Jönköping, Sweden. The system map illustrates the complexity of a real health system department. This example includes 81 discrete processes. Clusters of related processes are organized in dashed boxes.
  At the core of the system map are the mainstay processes that accomplish the organization's mission. A dark black square in Figure 2 emphasizes the mainstay. Read from left to right, the processes reflect the patient's journey and experience. For example, process #32 is triage patients in the [emergency room].
  On the top of the diagram are driver processes: planning, market research, and improvement. Here, processes are read from right to left, reflecting how information flows from the customer to improving and operating the system. The processes to the right focus on learning from customers and gathering information about meeting the need, which then feed into planning and design and redesign. For example, process #13 is collection of facts through literature and participation in conferences.
  Underneath are support processes that enable the mainstay to execute the delivery of products and services to customers and clients. Support processes do not follow a flow and are often organized beneath the mainstay processes they support. For example, process #79 is ordering supply.
  *Vector of measures*. Managing and improving an organization requires learning from measurement that provides multiple views of the organization (25). An organization's vector of measures includes select outcome measures displayed together in Shewhart charts (26). The components of the vector of measures relate to the organization's purpose and each measure depicts the story of a specific area of interest.
  Together, the collection of measures allows leaders to evaluate the performance of the organization. The charts are displayed together to enable seeing the system and appreciating the interdependencies within the organization. The vector is used as an indicator of current performance, to learn from special causes of variation (27), to see the impact of improvement efforts, to appreciate how key parts of the system are interdependent, and to make predictions about future performance.
  Figure 3 is the vector of measures displayed in Shewhart charts for a large ambulance service serving Mecklenburg County, North Carolina (28). Note many measures like patient and employee satisfaction (first row, first and second charts from the left) are stable with only common cause variation. There is also special cause like a shift of improvement in total task time (second row, third chart from the left), but a shift in an undesired direction in hours lost to on-the-job injuries (OJI) (bottom row, second chart from the left).
3. **Building a system for obtaining information**—Organizations have many systems in place to collect quantitative and qualitative data (29). This information should be systematically collected, analyzed, reported, and acted on, as appropriate. The aim of a system for obtaining information is to gather information about the need the organization is serving. Information comes from diverse sources, including patients, employees, and other internal and external sources.
  Figure 4 is a conceptual diagram showing a system for gathering information. It begins with the appreciation of the need the organization intends to fulfill with its services and an understanding of the dimensions of quality that are important to the customers. Information is collected from diverse sources (people and internal and external means) and matched to actions: problem-solving, improvement, and analyzing for future planning (11).
  Baptist Memorial Health Care provides an example of a source of information gathering. Quick response (QR) codes are placed on its units (Skip Steward, personal communication, 30 September 2025). If anyone—patients, family, and staff—sees something to be fixed (for example hand sanitizer stations running low), they can scan the QR code with their smartphone. A web app opens allowing the person to submit a picture and information. A commercial product links the submission to the unit and routes it to the responsible leaders for action. Data are also aggregated into a dashboard.
  Equally important to gathering the data is to organize and analyze it to enable learning about opportunities for improvement. As data are collected, they are related to the system through a specific process, product, or service. This is designed to enable immediate action to solve a problem in the organization and provide feedback for existing improvement efforts.
  In addition, data are analyzed and prepared to serve as input into the organization's planning activities. For example, data from customer comments are reviewed during the year and categorized for display in a Pareto chart. The chart and related data are analyzed and become an input to the planning process.
4. **Planning to improve**—Continuous improvement of an organization requires selecting efforts that are strategic for the organization to fulfill its mission and ensure that its services continue to match the need (30). Planning requires a method to gather inputs, process the information, and relate it to improving the system.
  Figure 5 is a conceptual diagram of planning as a system (11). On an ongoing basis, leaders are learning from both operating and improving the system (upper right). Inputs are summarized annually including the purpose, system map, vector of measures, and other information for leaders to study in advance (upper left). Moving from left to right, leaders study the inputs and develop or update strategic objectives based on themes that are revealed. How does the organization achieve these strategic objectives and prioritize and predict impact? By relating the strategic objectives to the organization using the system map, the required processes and services are identified where change is required. The vector of measures also helps to predict whether changes will affect the measures of interest indicating progress on the stated strategic objective. This results in a list of impactful opportunities that either becomes part of a portfolio of improvement projects (plans to improve) or it informs plans to operate the organization. Plans to improve become chartered and resourced projects and plans to coordinate with the organization's other planning and budgeting process serve to guide in operating the organization.
  Improvement Cymru is a national improvement organization that provides training and commissions and organizes improvement efforts across Wales. During the planning process, leaders related their strategic priorities to their system map. Several processes and services were identified as critical to achieving results. Many of these processes were previously rated as needing definition to standardize practice and stabilize outcomes. The planning process enabled identifying where in the organization change was required to design or redesign their work to achieve their stated goals. Their system map and the rating of each process condition supported pinpointing where to act and recognizing the gap that must be reduced to achieve their aims. The planning process focused the leaders to plan a portfolio of improvement projects directly aligned with their strategic objectives.
5. **Managing improvement efforts**—Healthcare organizations today typically have some improvement capability and active improvement projects (31). Results-driven improvement that achieves the strategic objectives of the organization is central to adopting quality as a strategy for the organization. Leaders must adopt a shared method like the Model for Improvement (Figure 6) (29) and build capability for people and teams to design and execute projects that contribute to accomplishing the purpose of the organization. Leaders also must develop methods and routines (32) to manage a portfolio of improvement efforts and sponsor (33) teams to both help remove barriers and learn about the organization.
  Supplementary Figure S1 is an illustrative example of the journey of a portfolio of improvement efforts by tracking an organization's project progress scores (on a 1–10 scale) over time (11). Project 4 has not moved to testing changes and the leader serving as sponsor can connect with the team to learn more and spur progress. As projects are completed, progress reports help leaders understand what is required to change the system, and then new projects are chartered for the next cycle. These improvement efforts are key to achieving the business plans and strategic objectives of the organization that were developed by the leadership team.

In addition to the five activities depicted in Figure 1, equally important are the arrows that show the interdependence of the activities and how they relate together as a system. For example, the mission defines the mainstay processes of the organization. The activities are not isolated or independent but integrated. Efforts to design and redesign the organization using a shared method and learning by applying tools to understand the system, make changes, and see the impact with measurement are central to improvement science and the theory of knowledge.

**Table 1:** Methods for leaders to focus the organization on improvement.

Methods	Description of actions for making quality an organizational strategy
**Purpose** **activity**	**Establish and communicate the purpose of the organization** Develop a written statement of purpose for the organization, including the mission, tenets, and vision. Communicate this purpose to the organization by relating the work of different parts of the organization to the purpose. Document connection of purpose with the role statements for departments and all employees. Use this purpose to guide and focus the organization as it conducts business, makes decisions, and manages improvement.
**System** **activity**	**View the organization as a system** Understand the major processes and products/services in the organization. Document how these processes link together to form a system. Use the system map of the organization to understand the work and focus of improvement efforts. Create a vector of measures to understand the performance of the system. Visually display these measures as a set of Shewhart charts to see the system's performance. Use these documents to understand the impact of improvement on the organization and to learn how the organization functions as a system.
**Obtaining information** **activity (customer** **focus)**	**Establish a system to obtain information relevant to the need that the organization is fulfilling** Identify the present and future customers of the organization. Develop a system to gather information about matching the need. Develop systems to obtain other information relevant to the need. Communicate this information to all parts of the organization. Analyze this information to guide planning and improvement efforts.
**Planning** **activity**	**Planning to improve** Summarize the information from customer research and from employees, suppliers, and the relevant external environment. Based on these inputs, develop (or update) strategic objectives that could best accelerate the performance of the organization. Develop a list, in order of priority, of the processes, products, and services to design or redesign. Coordinate this plan with the organization's strategic and business planning and budgeting activities. Establish briefs for improvement projects that can be resourced and managed.
**Managing improvement activity**	**Manage improvement efforts** Prepare the organization to focus on improvement. Define the leadership team's role in managing improvement efforts. Execute improvement projects identified in the planning process. Provide a standard methodology to guide improvement efforts. Provide training and other necessary resources required for improvement efforts. Provide sponsors and ensure that guidance is provided for improvement efforts. Remove obstacles and provide recognition. Redirect and redeploy resources as improvements are made. Leadership team studies the improvement results and contributions of the improvement efforts of the team to learn about the organization viewed as a system and the key forces driving the system.

**Table 2:** Purpose statement showing the mission describing the need, vision, and core values (Cincinnati Children's Hospital Medical Center, Cincinnati, Ohio, USA).

Component	Organization's example
Mission	Cincinnati Children's will improve child health and transform the delivery of care through a fully integrated, globally recognized research, education, and innovation. For patients from our community, the nation and the world, the care we provide will achieve the best: Medical and quality-of-life outcomes Patient and family experience Value today and in the future.
Vision	Cincinnati Children's will be the leader in improving child health.
Core values	Compassionate—we are champions of diversity, equity, and inclusion, supporting everyone with care, recognition, and intentional development. Collaborative—we strive to get to the best answer by working as one Cincinnati Children's. Honest—we lead with integrity, courage, accountability, and high ethical standards. Impactful—we drive to improve outcomes, experience, and value for kids, families, teams, and the community. Curious—we embody a mindset of creativity, innovation, learning, and discovery.

### Getting started

Leaders choose where it is appropriate for their organization to start pursuing quality as a strategy. Results-driven improvement capability is central to this work (34). Focus begins on a portfolio of improvement projects that are currently active or new ones that are started. Leaders serve as sponsors, and together with the teams, the projects become learning labs to understand how to execute improvement and achieve the desired aim and to learn about the organization, its culture, and the friction that hinders progress.

In parallel, the leadership team revisits the organization's purpose and how it is communicated and used and begins to develop a learning system. Understanding the current state of each of the QOS activities follows and, as the organization uses these methods, they develop them, improve them, and link them together (35).

The journey to pursue QOS includes three phases: Developing, Using, and Understanding. As leaders build the system, they reach milestones along the journey. Supplementary Table S1 lists the three phases of the journey and milestones achieved along the way (11).

Improvement projects and the journey to learn about the organization as a system through the activities invite people to learn about themselves, each other, their work, and their organization. In our experience, when people use methods to learn deeply about their work together and can make changes to the system that result in both qualitative and quantitative improvement, they are motivated, engaged, and show pride in their work. This experience is the final link to improvement science and psychology and human behavior.

## Case study—large ambulance service

The following case describes examples from each of the five QOS activities just described from a healthcare organization pursuing quality as an organizational strategy. The case is presented including some examples of the process and learning. It does not include all the details and artifacts of the organization's full work. It also does not aim to describe its journey chronologically.

Mecklenburg EMS Agency is the ambulance service responsible for 9-1-1 medical communications, emergency ambulance, and interfacility medical transport in Mecklenburg County and the City of Charlotte. The organization's communication center and ambulance services are accredited and the organization's performance benchmarks well nationally.

Over a 2-year period, the leadership team pursued QOS with support from the authors and progressed through the first 17 milestones of the development phase. The leaders began by better understanding the need that the organization was intending to fulfill and reviewing their purpose and how it was used and communicated. Next, they developed a system map; first conceptually and then a detailed version including all processes and their linkages. In the second year, they developed the inputs for planning and participated in the planning activity. Concurrently, improvement projects were active using the Model for Improvement. Leadership and staff continued the work independently, without external support.

### Establishing a purpose

The leaders reevaluated the need that the organization's services were intending to fulfill, the dimensions of quality that mattered to their patients and community, and they considered how they were using their tenets in practice. Like other organizations, they discovered opportunities to clarify their purpose and consolidate versions that existed in different documents (35). The purpose statement was updated and communicated to the organization and the leadership integrated the tenets into the decision-making process.

### Viewing the organization as a system

The leadership initially created a conceptual view of the organization. This was followed by listing the mainstay, driver, and support processes. A process boundary form was produced for every process outlining what was required to start it and from whom, the start and end points, and what the desired outcome of the process was and for whom. If an agreed upon process or procedure existed, that was noted too. The processes were organized visually into a system map depicting the core processes and how they were believed to link together.

Each task resulted in deep learning about the organization and how it worked. For example, processes were discovered to be lacking in standard methods, documentation, and defined measures to know whether they were effective. Also, some processes were determined to not be fit for purpose and using resources that could be reassigned to other responsibilities. For example, routine reporting was discovered to include data not used by the leaders receiving the reports. Updating the reporting to match the requirements of the receiving leaders reduced task time in the process.

The organization had a vector of measures displayed together in Shewhart charts but identified a need to document operational definitions and ensure proper data collection. For example, a key business process measure for the fleet was interpreted differently by staff capturing the data and the leaders making decisions based on the measure chart.

### Information

The organization possessed several independent sources of information, including customer and staff surveys, focus groups, complaint mechanisms, and data from various systems. Leadership identified the sources and created methods to summarize and direct information to responsible management and staff and for the planning process. A new process included each leadership team member directly engaging suppliers to learn how their partnership could improve the organization, resulting in discussions to improve the preventive maintenance of their cardiac monitors and stretchers.

### Planning to Improve

In preparation for the annual planning, leadership prepared a planning packet with a diverse set of inputs. The leadership team and board members reviewed the packet in advance of the planning event. The event followed an adapted version of the QOS planning process, resulting in agreement on a portfolio of improvement projects and plans to operate. The process focused the planning process on improvements directly related to their strategic objectives and highlighted where resource adjustments in operations were needed. Leaders relayed that the participants reached agreement quickly and were able to select priorities together because of the preparation and studying of the inputs before engaging in the planning process.

### Managing Improvement

**Figure 7:**
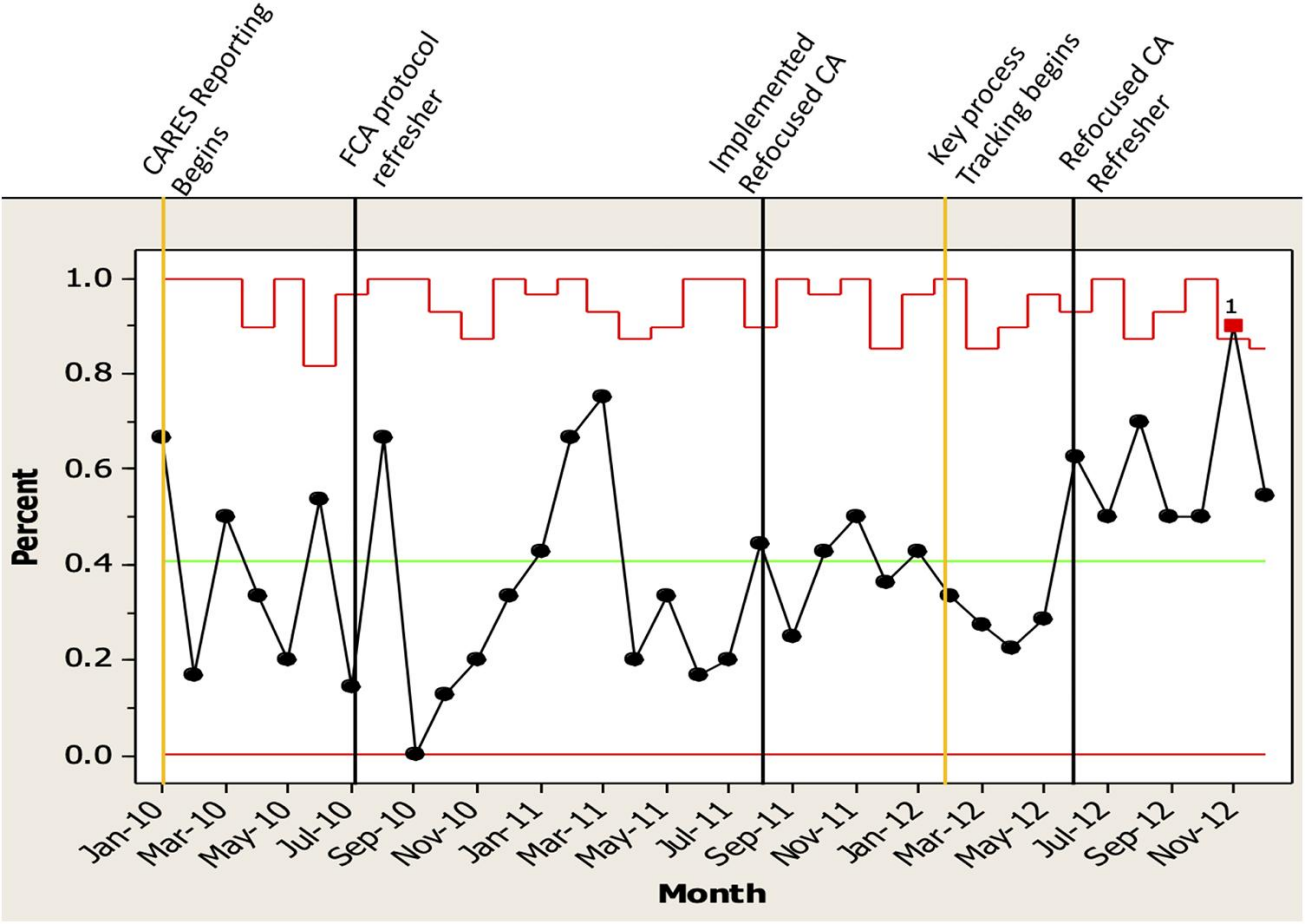
Shewhart chart (P chart) of the outcome measure of an improvement effort to increase community survival rates from a witnessed cardiac arrest with process changes annotated. Reprinted with permission from Medic (Mecklenburg County EMS Agency), Keith J. Permission granted 25 February 2026.

The organization adopted the Model for Improvement and used it to charter and execute improvement projects. Staff continued to build on their ability to execute results-driven improvement. For example, the leaders used the Model for Improvement to benchmark with another organization known for achieving the highest sustained out-of-hospital sudden cardiac arrest survival outcomes. They distilled the key drivers and developed a family of measures for the project. Several sequential PDSA cycles resulted in a system-wide implementation of changes to their approach to resuscitation that correlated with a shift in their survival rate to 50 percent, which matched the top national survival rate and sustained it over time (36). Figure 7 is a time series Shewhart chart (P chart) with annotations describing the changes tested.

### Case study summary

Mecklenburg EMS Agency's journey through the QOS development phase demonstrates the integrated nature of the five activities. The organization refined its purpose, created visual representations of its work (system map) and performance (vector of measures), established mechanisms for gathering and using information, embedded these inputs into strategic planning, and built capability for results-driven improvement. The cardiac arrest improvement example illustrates how managing improvement efforts produces both results (improved survival rates) and organizational learning about system performance. This 2-year development phase established the foundation for ongoing use and understanding of QOS as the organization's approach to continual improvement.

## Anticipated results

QOS is a results-driven improvement and learning system (34). Learning how to execute improvement projects and achieve aims at an efficient and predictable pace (29) is essential to building a habit of improvement (37) for the organization. The organization builds toward improvement that makes progress on strategic objectives and enhances organizational performance as described by their vector of measures. The theories and methods of improvement science become a norm in the organization.

### Assessing progress and impact

QOS includes several methods to support leaders in monitoring progress along the journey. Leaders can individually evaluate their progress across ten areas of QOS with an assessment tool (Supplementary Table S2) (11). Individual assessments are combined to learn the average score and the variation within the scoring. Reassessment occurs at regular intervals. QOS includes three phases: Developing, Using, and Understanding. As leaders build the system, they reach milestones along the journey. Supplementary Table S1 lists the three phases of the journey and milestones achieved along the way (11).

Self-assessments can reflect bias and variation across leadership team members. The rubric definitions are crafted with specificity to aid the assessment. Typically, leaders assess higher on their initial assessment and then correct their assessments as they engage in the work and better understand the current state of their organization. The method invites discussion as to why different ratings were chosen and to together reach a consensus score. Disagreement can be redirected to running a PDSA for gathering information to learn from varying assessments.

Formative evaluation may include several measures of improvement (38). A project progress scale provides a rubric to evaluate project progression (11). The family of measures of each project displayed in time series Shewhart charts are used to assess special cause as changes are tested and implemented in pursuit of the aim (27). A process condition rubric is used to assess the current condition of each process on the system map (11). When improvement efforts are chartered and executed, the process condition scores on the system map should achieve a score of 1 or 2. Measures in the vector related to the strategic objectives will likely see improvement as successful improvement projects are completed and implemented. Finally, a tool like the MUSIQ scale (39) offers insight into the ability of organizations to do improvement work.

## Discussion

QOS builds on the foundations of improvement science. It is designed to align improvement efforts to the organization's purpose and their customers, focus on results-driven improvement, continually improving across the system, and engage people in a learning organization. It evolves from the current state of project-based improvement and capability efforts in healthcare. It helps integrate the methods in a system of activities working together. This integration distinguishes QOS from an isolated application of improvement tools. Each activity draws on and reinforces the foundational bodies of knowledge in improvement science, creating a coherent system for organizational transformation. QOS is generalizable to varied organizational contexts and can be replicated using its five activities for leaders and the methods described.

Large (thousands of staff) and small (less than 50 staff) health and healthcare organizations including hospitals, ambulance services, public health organizations, and improvement organizations have pursued QOS. Organizations in other industries such as business, education, manufacturing, and service organizations have used the method including one Malcolm Baldrige Excellence Award recipient (40). QOS can be applied in any organization; the level of complexity will mirror the size, scale, and breadth of the organization. Formally evaluating the implementation of QOS in individual organizations beyond the process and clinical outcomes identified during QOS data collection will build the empirical evidence base for the method alongside identifying key factors that generate benefit.

Leadership gauges their organization's current progress using the QOS assessment. The work of leaders begins where they are and starts with improvement work on a portfolio of projects. In parallel, a leadership learning system develops that teaches the theories of improvement science and the QOS activities and supports applying the theory to practice. Leaders continue the journey with understanding, improving, and communicating the organization's purpose.

### Practical and cultural challenges

Pursuing QOS is not without practical and cultural challenges. First, leadership is responsible for the organization and plays a central role in facilitating attention, resourcing, and change. If leaders are not engaged and leading, QOS will not thrive. Second, developing and using QOS includes many activities that may be new or require different methods that take time, effort, and discipline. There is no universal toolset to develop and use. Like improvement projects, the work of QOS can start, stop, and start again as leaders and staff learn and try new methods (41).

The role of will, ideas, and execution is familiar in improvement work and apply in QOS as well (42). Improvement of the system is essential to successful implementation to avoid the activity trap and build the engagement of staff. Staff are motivated when they are actively involved in improvement efforts that enable understanding and changing their work processes, reducing friction, and improving the results that they are trying to achieve for patients (43, 44).

### Opportunity to learn across implementations

The methods of QOS are integrated into familiar places, but they are represented as business as usual. For example, the learning health systems model builds on the methods described in QOS (45), as does the management model taught to the leaders in Jönköping, Sweden (46). There is an opportunity to learn from variations of adoption and to compare the journeys, progress, and lessons learned. For example, learning health networks use a maturity model like the QOS assessment and have published comparisons of their self-assessed progress using their rubric (47). Organizations should also share lessons learned. For example, NHS Wales published insights from their own efforts to implement QOS such as “focus on system and process design … shifted the quality conversation and approach” and “use outputs that are ‘good enough’ and iterate as you learn from using [it]” (48)

### Execution and implementation

Leadership is central to adopting the methods across the organization. Leaders must be engaged and create the space to participate. Without their leadership and participation, QOS will never mature. Often a leader serves as the steward of the process. This can be a chief quality officer or other member of the leadership team. The impact of QOS is realized through action. There is a risk when leaders approach QOS as professional education and exercises and do not apply the methods in practice in the organization. Improvement projects using tools and methods with fidelity and achieving their aims are essential skills in QOS. An active portfolio of improvement projects sponsored by leaders should always be part of the approach. They serve as a vehicle for learning and understanding the organization's practices and culture and in effecting change in the system.

### Limitations

QOS is not simple, nor are healthcare organizations. QOS requires leadership to apply methods with which they may not have experience across their organizations. QOS must be led and managed by the leaders of the organization; commitment and participation are required. The journey depends on engagement across staff groups. Results-driven improvement can be hard work. Building a system of improvement and learning across a complex organization is a difficult task for leaders. The journey takes an ongoing effort over years and requires developing new approaches, routines, and learning by the leadership team.

A common hindrance to progress is the degree of leadership engagement in improvement and developing the system. Organizations that overestimate their improvement capability often want to jump into QOS activities. Enhancing the ability to systematically achieve results on projects must occur in parallel to building the system. QOS activities are not independent; separating or siloing work will not fully realize the potential of adopting quality as a strategy.

The process is iterative and unfolds over time, which requires discipline in the design and execution of the approach. The five activities described previously include several components and building the system evolves with use and focus. Leadership transition can also alter, slow, or halt progress.

Organizations may encounter contextual barriers when implementing the framework. Factors such as organizational culture, leadership turnover, competing priorities, and sustainability of improvement initiatives can significantly influence the success of organizational change efforts.

### Future research

Case studies of organizations that have pursued QOS can serve as a fertile source of learning about the strengths and limitations of pursuing QOS. Organizations currently developing quality as their strategy or getting started should be encouraged to include documentation and dissemination of their progress, the methods, and the learning along the journey. A pilot of several organizations in a learning health system together or a large health system with multiple hospitals each pursing QOS and sharing their data and experience together would be a powerful mechanism to better see QOS adoption across an entity and learn about implementation under different conditions. For example, learning health systems can share their maturity model framework and can collaborate across networks to make progress in shared elements.

Further study of the conditions that support adoption and the lessons learned from developing the activities would reduce the friction and unknowns for leaders interested in pursuing quality as their strategy in the future. In addition, a formal evaluation of QOS as a method for generating system improvement is likely to generate more valuable learning about the method and its generalizability.

## Conclusion

Healthcare organizations are complex organizational systems. Improvement science and methods applied to project-based improvement are in use in many healthcare settings. There is interest in how these theories and methods can be applied across a system. QOS is a practical yet sophisticated approach for building a system of improvement at the organizational level. QOS builds on the theories of improvement science and adopts quality as the strategy of the organization. The approach is not quick or easy and requires commitment, discipline, and persistence to develop and use. QOS contributes to the frontiers of improvement science through system-level integration of theory and practice, offering methods to transform how leaders guide organizations and achieve results.

## Data Availability

The original contributions presented in the paper are included in the article/Supplementary Material; further inquiries can be directed to the corresponding author/s.
