# Supplementary material for: Quality as an organizational strategy: building a system of improvement
Source: Front Health Serv. 2026 May 29;6:1726688. doi: 10.3389/frhs.2026.1726688 (PMC13260625; doi:10.3389/frhs.2026.1726688)
Supplement: Supplementary file 1 [file Table1.pdf]

Supplementary Table S1. Milestones for Quality as an Organizational Strategy (QOS)

Phase 1<br>QOS development	Phase 2<br>Using the QOS system	Phase 3<br>Understanding QOS
1. Establish formal improvement efforts with individuals and teams.<br>2. Begin leadership education.<br>3. Recognize purpose and publish purpose statement.<br>4. Leaders begin delivering awareness education sessions.<br>5. Develop a view of the organization as system.<br>6. Establish key vector of measures of the system.<br>7. Identify system for customer research.<br>8. Formal improvement effort is successful.<br>9. All leaders participate in quality improvement effort.	10. Use the purpose statement in organization.<br>11. Use the systems map in the organization.<br>12. Use key measures in reports.<br>13. Analyze and condense customer feedback and research.<br>14. Leaders learn from improvement efforts of teams and individuals.<br>15. Planning drives improvement efforts.<br>16. Business plan integrates improvement.<br>17. Improvements are made to a major management system.<br>18. Involvement suppliers in improvement.<br>19. Involvement customers/partners in improvement.<br>20. A system is in place for developing capability in the Science of Improvement.<br>21. Reflect and recognize major improvements in the maturity of QOS.<br>22. Role statements connect all employees to the system.<br>23. Improvements are understood in terms of the Chain Reaction.	24. Leaders use Model for Improvement in decisions for learning.<br>25. Constancy of purpose is understood.<br>26. Leaders have a systems view.<br>27. Leaders understand variation.<br>28. All employees are listening, observing, and reporting what they learn.<br>29. All business planning process are fully integrated with planning for improvement.<br>30. Concepts of improvement are incorporated in the business infrastructure.<br>31. Everyone is involved in improvement.<br>32. Improvement philosophy, methods, and tools are used in day-to-day work.<br>33. Social change is understood as it relates to changes in psychology.<br>34. The Science of Improvement is used to develop and make changes.

Reference

Norman CL, Provost LP, Williams DM. Quality as an organizational strategy: Building a system of improvement. Austin, Texas: Provident-Heierman Press; 2024. 365
