# Supplementary material for: Quality as an organizational strategy: building a system of improvement
Source: Front Health Serv. 2026 May 29;6:1726688. doi: 10.3389/frhs.2026.1726688 (PMC13260625; doi:10.3389/frhs.2026.1726688)
Supplement: Supplementary file 2 [file Table2.pdf]

Supplementary Table S2. Assessment of progress in making Quality an Organizational Strategy (QOS)

	**Operational definition and scoring**					
**Activities**	Just Beginning (Score = 0)	Aware (Score = 2)	Informed (Score = 4)	Integrating (Score = 6)	Understanding (Score = 8)	Successful and renewing (Score = 10)
Purpose	No written statements	Statement exists	Mission and tenets defined and visible	Communicated and understood by employees	Used to align and guide the organization	Fully integrated into the structure
Organization as a system	Work as a process is not understood	Major processes are documented	Relationships between processes are documented	Systems thinking and language are common	Systems diagrams are used in the organization	Management systems have integrated the systems view
System measures	Financial data are used for management reports	Financial and other operational measures are used	Family of measures is assembled and reported regularly	Balanced set of measures, each presented as a time series	Set of measures aligned; both variation and interrelationships are understood	Set of balanced measures fully integrated into all management systems
Information	Information is gathered on ad hoc, reactive basis	System is based on passive information	System is well documented and includes active sources	Information is documented and communicated	Comprehensive system with analysis and synthesis for decision making	Marketing leads and integrates information system
Planning for improvement	No formal long-term planning, reactive culture	Planning for improvement is done on an informal basis	A formal, documented process exists for planning improvement	Integrated process identifies objectives, efforts, and resources	All other planning processes are defined and linked with planning to improve	Planning system is regularly improved and integrated in all areas
Managing improvement efforts	No system exists to manage improvement efforts	Improvements recognized on an as-needed basis and resources assigned	Leaders provide formal guidance for individuals and teams	Improvements are guided by planning; leaders learn from all improvements	The impact of improvement is understood and actively managed to achieve benefits	Improvement system is integrated in and continuously improved
Model for Improvement	No standard approach to improvement efforts	Various approaches are used for improvement	Training on the model and expectation of its use	Theory behind the model is understood	Improvements are managed as PDSA cycles	Model for improvement is routinely used by all
Leadership system	Structure does not exist to make improvement a focus of the organization	The importance of improvement is recognized, and responsibility assigned	A formal system for improvement is defined	Leadership team assumes responsibility for integrating improvement	Improvement is linked to planning and other key business activities	Improvement is completely integrated into all aspects of operating and developing the business

Reference

Norman CL, Provost LP, Williams DM. Quality as an organizational strategy: Building a system of improvement. Austin, Texas: Provident-Heierman Press; 2024. 33
