# Supplementary figures and images for: Quality as an organizational strategy: building a system of improvement
Source: Front Health Serv. 2026 May 29;6:1726688. doi: 10.3389/frhs.2026.1726688 (PMC13260625; doi:10.3389/frhs.2026.1726688)

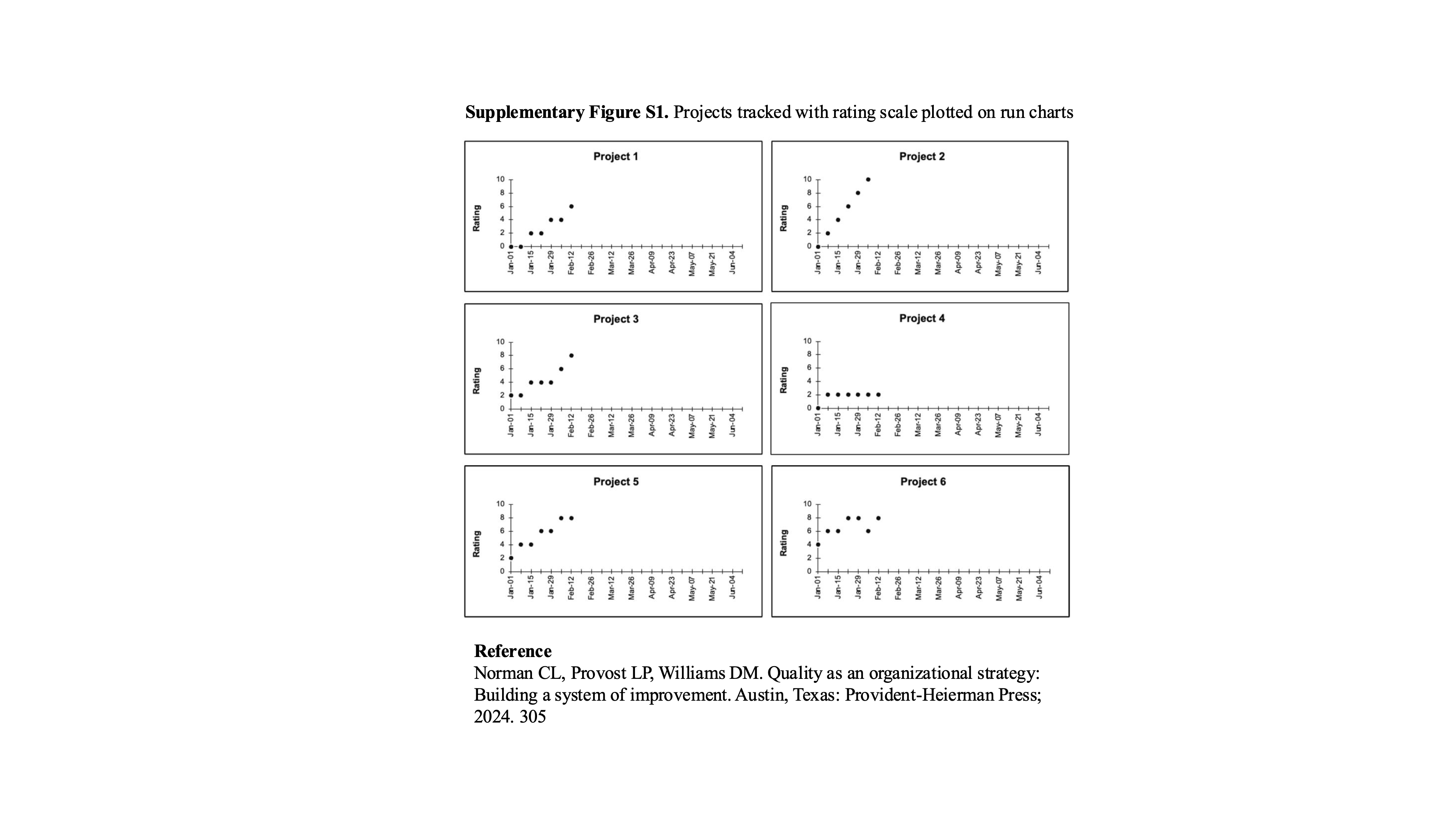

Supplement: Supplementary file 3 [file Image1.tiff]
